# Inhibition of the vacuolar ATPase induces Bnip3-dependent death of cancer cells and a reduction in tumor burden and metastasis

**DOI:** 10.18632/oncotarget.1699

**Published:** 2013-12-29

**Authors:** Regina M. Graham, John W. Thompson, Keith A. Webster

**Affiliations:** ^1^ Department of Molecular and Cellular Pharmacology and the Vascular Biology Institute, University of Miami School of Medicine, Miami, FL; ^2^ present address: Department of Neurological Surgery, Miller School of Medicine, University of Miami. Miami, Fl; ^3^ present address: Department of Neurology, Miller School of Medicine, University of Miami. Miami, Fl

**Keywords:** hypoxia, cancer, xenograft, programmed cell death, bafilomycin

## Abstract

The pro-apoptotic protein Bnip3 is induced by hypoxia and is present in the core regions of most solid tumors. Bnip3 induces programmed necrosis by an intrinsic caspase independent mitochondrial pathway. Many tumor cells have evolved pathways to evade Bnip3-mediated death attesting to the physiological relevance of the survival threat imposed by Bnip3. We have reported that acidosis can trigger the Bnip3 death pathway in hypoxic cells therefore we hypothesized that manipulation of intracellular pH by pharmacological inhibition of the vacuolar (v)ATPase proton pump, a significant pH control pathway, may activate Bnip3 and promote death of hypoxic cells within the tumor. Here we confirm that bafilomycin A1 (BafA1), a selective vATPase inhibitor, significantly increased death of breast cancer cells in a hypoxia and Bnip3-dependent manner and significantly reduced tumor growth in MCF7 and MDA-MB-231 mouse xenografts. Combined treatment of cells with BafA1 and the ERK1/2 inhibitor U0126 further augmented cell death. Combined treatment of mice containing MDA-MB-231 xenografts with BafA1 and the ERK1/2 inhibitor sorafenib was superior to either treatment alone and supported tumor regression. BafA1 and sorafenib treatments alone reduced MDA-MB-231 cell metastasis and again the combination was significantly more effective than either treatment alone and was without apparent side effects. These results present a novel mechanism to destroy hypoxic tumor cells that may help reverse the resistance of hypoxic tumors to radiation and chemotherapy and perhaps target tumor stem cells.

## INTRODUCTION

Most solid tumors are characterized by regions of transient hypoxia that frequently correlate with poor clinical prognosis [1-3]. The hypoxic regions result from a high oxygen demand of rapidly dividing cells at the tumor periphery combined with a disorganized vasculature [4, 5]. The hypoxic zones typically boarder a necrotic core that is deprived of blood supply [6, 7]. Tumor hypoxia contributes to chemo and radiation therapy resistance and generates a more malignant, metastatic phenotype by favoring increased rates of mutagenesis, up-regulating the expression of multidrug resistance genes and selecting for cells with mutated p53 [8-10].

The rate of glycolysis, already high in many aerobic tumor cells, is increased even more in hypoxic regions resulting in elevated lactic acid production and acidosis, a situation that is further compounded by inefficient vascular/lymphatic clearance of metabolic waste products [11-13]. Tumors typically maintain a neutral intracellular pH by elevated activity of carbonic anhydrase and aggressive extrusion of protons [14-16]. The latter is accomplished by the activity of at least four major ion channels including the vacuolar proton pump (V-ATPase), the Na+/H+ exchanger, the bicarbonate transporters and the monocarboxylate transporters [17, 18]. The vacuolar ATPase belongs to a family of proton pumps associated primarily with endosomes but present in the plasma membrane in cancer cells [19, 20]. Pharmacological inhibition of the vacuolar ATPase activity inhibits autophagy and may promote apoptosis by reducing the pH buffering capacity and promoting intracellular acidosis [21].

Bnip3 is an atypical member of the BH3-only subfamily of proapoptotic proteins with possible roles in programmed cell death and autophagy, and myocardial and neuronal ischemia/reperfusion injury [22-24]. Bnip3 is a hypoxia-regulated gene and its expression is low to absent at normal physiological oxygen tension but increases dramatically upon exposure to hypoxia/ischemia [25]. Co-expression of Bnip3 and HIF-1α has been demonstrated in multiple types of tumors including prostate, lung, and endometrial [26-28]. More than 70% of invasive breast cancer and 60% of ductal carcinoma in situ (DCIS) express Bnip3. In non-small cell lung carcinoma, high levels of Bnip3 expression are associated with poor clinical outcome and in prostate cancer Bnip3 expression strongly correlates with the cancer stage or “Gleason” score [26, 28].

The identity of Bnip3 as a positive prognostic indicator of tumor progression appears paradoxical with its role as a pro-apoptotic mediator, and some have concluded that Bnip3 can actually provide a survival function by increasing reparative autophagy [29, 30]. Alternatively, our group has proposed that Bnip3, accumulated during hypoxia is inactive and the death function requires a second activation step such as coincident acidosis [31, 32]. This appears to be the case at least in striated cardiac and skeletal myocytes where hypoxia alone does not cause cell death despite marked elevations of Bnip3 33. In contrast massive Bnip3-dependent cell death is seen when hypoxia is combined with acidosis, a condition frequently associated with chronic hypoxia or ischemia [32]. The death pathway mediated by Bnip3 under conditions of hypoxia-acidosis is characterized by the opening of the mitochondrial permeability transition pore, release of cytochrome c, calpain but not caspase activation and extensive DNA fragmentation [25, 33, 34]. Because the Bnip3 death pathway is so aggressive, we were interested to determine whether it could be induced in hypoxic breast cancer cells by blocking one of the major proton ejection channels thereby disrupting the intracellular pH buffer capability. To this end we found that inhibition of the V-ATPase with bafilomycin A1 (Baf1A) indeed activated the Bnip3 death pathway in vitro and in vivo. We found that cell death by this mechanism was enhanced by simultaneous inhibition of the ERK1/2 signaling pathway. Using xenograft models we demonstrate that the combination of Baf1A with the ERK inhibitor, sorafenib, significantly improved treatment efficacy causing both tumor regression and inhibition of metastasis.

## RESULTS

### Bafilomycin A1 (Baf1A) induces cell death and causes Bnip3 stabilization

**Figure 1:**
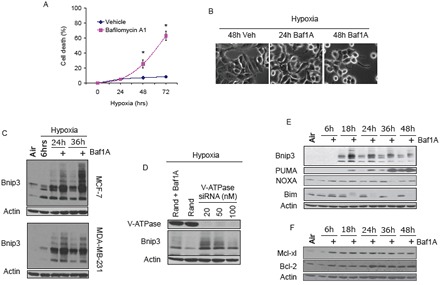
Vacuolar ATPase inhibition induces Bnip3 dependent cell death MCF-7 cells were exposed to hypoxia for times indicated in the presence and absence of Baf1A. The effects of Baf1A on cell death was determined by trypan blue exclusion in (A) and on cell morphology in (B). The effects of Baf1A on Bnip3 protein expression in both MCF7 and MDA-MB-231 cells is shown in (C). In (D), MCF7 cells were treated with random sequence or vacuolar ATPase specific siRNA and the level of Bnip3 protein expression determined after 36 hrs of hypoxia. As a positive control a parallel plate was treated with Baf1A in the presence of random sequence siRNA (D). Expression of BH3-only proapoptotic proteins (E) and anti-apoptotic proteins (F) during hypoxia with and without Baf1A. Data are means ± SEM. * *p* < 0.05 compared to vehicle treated samples. All results are representative of at least 3 experiments.

Previously we reported that acidosis combined with hypoxia conferred enhanced stabilization and accumulation of Bnip3 relative to hypoxia at neutral pH [31]. Therefore we asked whether inhibition of the V-ATPase in cancer cells under hypoxic conditions supported a similar stabilization and accumulation of Bnip3 by conferring intracellular acidosis. As shown in Figure 1, exposure of MCF7 and MDA-MB-231 breast cancer cells to hypoxia caused a progressive increase in Bnip3 protein that started at 6 hrs of hypoxia and peaked at 24 hrs. Despite induction of Bnip3 we observed no cell loss during 72 hrs of hypoxic exposure. However, when MCF7 and MDA-MB-231 cells were treated with the V-ATPase inhibitor Baf1A before exposure to hypoxia, Bnip3 protein levels were significantly increased relative to hypoxia alone and there was a significant increase in cell death (65 ± 8.5%; *p* < 0.05; verses untreated controls) at 72 hrs. A similar increase in Bnip3 protein was observed when MCF cells were treated with V-ATPase specific siRNA (Figure 1E). To begin to address the mechanism of Baf1A toxicity we measured other BCL-2 family members and found no change in expression of the anti-apoptotic proteins Mcl-xl and Bcl-2 or NOXA, another BH3-only pro-apoptotic protein (Figure 1 F and G). In contrast Baf1A treatment dramatically affected the expression of the BH3-only pro-apoptotic proteins PUMA and Bim. PUMA protein levels were increased under hypoxia and, similar to Bnip3 were further increased by Baf1A whereas Bim expression was eliminated by Baf1A treatment. In experiments not shown we found that inhibition of the sodium bicarbonate transporter or the sodium hydrogen exchanger with DIDS and amiloride respectively were without effect on Bnip3 protein or cell death therefore the effects were selective for the V-ATPase (Data not shown).

**Figure 2:**
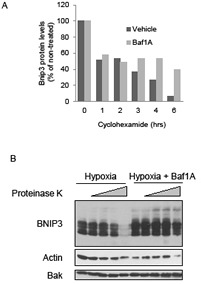
Bafilomycin 1A increases stability and decreases proteinase k susceptibility of Bnip3 The effects of Baf1A on Bnip3 protein half-life was determined by exposing hypoxic MCF7 cells to 5 μg/ml cycloheximide for the indicated times. Cells were harvested and the level of Bnip3 protein determined by western blot analysis (A). The susceptibility of Bnip3 protein to proteinase K digestion was determined in hypoxic MCF7 cells in the presence and absence of Baf1A. The cells were lysed and exposed to 0, 0.1, 0.5, 1.0, and 5.0 μg/ml proteinase k for 30 min. Bnip3 proteins levels were determined by western blot analysis (B). All results are representative of at least 3 experiments.

We have previously reported that the half-life of Bnip3 is increased by hypoxia-acidosis and this accounts significantly for the elevated levels of Bnip3 caused by acidosis [31]. To determine whether Baf1A treatment also increased Bnip3 protein half-life MCF7 cell were exposed to hypoxia alone or hypoxia plus Baf1A and protein translation was blocked with cyclohexamide. Western blot analyses revealed that Baf1A treatment increased the half-life of Bnip3 protein by 2.7 fold (n = 3) over hypoxia alone (Figure 2A). Previously we reported that acidosis reduced the susceptibility of Bnip3 protein to digestion by proteinase k suggesting a conformational change or membrane insertion in response to acidosis [31]. To determine if Baf1A conferred a similar decrease in Bnip3 proteinase k susceptibility, whole cell extracts were prepared from MCF7 cells exposed to hypoxia alone or hypoxia-Baf1A. The extracts were exposed to increasing concentrations of proteinase k and the level of Bnip3 protein determined by Western blot. As shown in Figure 2B, Baf1A treatment significantly reduced the susceptibility of Bnip3 to digestion by proteinase k. In contrast, digestion of actin by proteinase k was unaffected by Baf1A treatment and BAK, an integral outer mitochondrial membrane protein, was unaffected by proteinase k treatment under either condition. These results indicate that inhibition of the vacuolar ATPase increases Bnip3 protein stability possibly by promoting intracellular acidosis and driving membrane insertion as we have previously demonstrated [31].

### Baf1A induces Bnip3 mediated cell death

**Figure 3:**
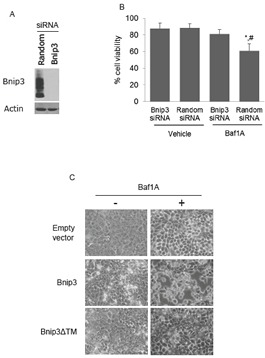
Bafilomycin 1A activates Bnip3 mediated cell death Western blot analysis of Bnip3 protein in cells treated with either random or Bnip3 specific siRNA (A). To determine if Baf1A induced cell death was dependent upon Bnip3, cells were treated with random sequence or Bnip3 specific siRNA in the presence and absence of Baf1A and cell death determined by trypan blue exclusion (B). In (C) the effects of Baf1A on normoxic MCF7 cell transfected with either empty vector, Bnip3 or Bnip3ΔTM (C). Data are means ± SEM. * *p* < 0.02 compared to Bnip3 siRNA treated samples; # *p* < 0.01 compared to Random siRNA/Vehicle. All results are representative of at least 3 experiments.

To determine whether Bnip3 is required for Baf1A-induced cell death we used Bnip3 specific siRNAs to knockdown Bnip3 protein. As shown in Figure 3A, Bnip3 expression was significantly reduced by Bnip3-specific but not random sequence siRNA. Treatment of hypoxia-neutral cells with Bnip3-selective or random sequence siRNA did not affect cell viability as assessed by trypan blue exclusion (Figure 3B). However cells exposed to Baf1A-hypoxia, sustained significant loss of viability which was ameliorated by Bnip3-selective siRNA (*p* < 0.02). To confirm that Baf1A can activate Bnip3 dependent cell death we over-expressed Bnip3 in normoxic cultures in the presence and absence of Baf1A. As shown in Figure 3C, Bnip3 expression alone was without effect on cell morphology relative to empty vector. In contrast, treatment of cultures with Baf1A caused cell detachment and rounding characteristic of cell death. The effect was abolished when Baf1A treated cells were transfected with a Bnip3 transmembrane deletion mutant (Bnip3ΔTM). The transmembrane domain of Bnip3 has been shown by ourselves and others to be required for Bnip3 mediated death [32, 35]. These results suggest that Baf1A activates the Bnip3 death pathway in hypoxic MCF7 cells.

### Pathway of Bnip3 mediated cell death

**Figure 4:**
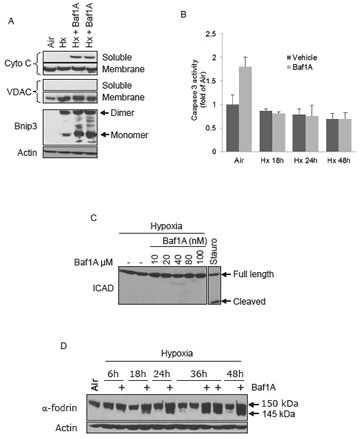
Caspase independent cell death MCF7 cells were exposed to air, hypoxia (Hx) or hypoxia with Baf1A for 36 hrs and the cells fractionated into soluble (cytoplasmic) and heavy membrane (mitochondrial) fractions. Cytoplasmic levels of cytochrome c is shown in (A). Fractional purity was determined by the mitochondrial marker VDAC. In (B) caspase 3 enzymatic activity was determined in MCF7 cells exposed to air (48 hrs) or to hypoxia (Hx) in the presence and absence of Baf1A for times indicated. The level of cleaved ICAD, a target of caspase 3, was determined in hypoxic MCF7 cells exposed to increasing concentrations of Baf1A (C). As a positive control a parallel plate was treated with 1 μM staurosporine, a potent inducer of caspase dependent apoptosis. In (D), proteolysis of the calpain target, α-fodrin, was determined in MCF7 cell exposed to hypoxia and hypoxia-Baf1A for indicated times. Data are means ± SEM. All results are representative of at least 3 experiments.

We have reported that cell death caused by hypoxia-acidosis is associated with the release of cytochrome c from the mitochondria but not caspase activation [32, 33]. Instead our results suggest that calpains are activated under these conditional and may be the central mediators of cell death. To determine if a similar death pathway is activated by the combination of hypoxia and Baf1A we used subcellular fractionation to measure cytochrome c release from the mitochondria. As shown in Figure 4A cytochrome c was not significantly present in the cytoplasmic fraction of aerobic or hypoxia-neutral cultures but the levels increased markedly in extracts of hypoxic cultures treated with Baf1A. Despite the apparent increased cytoplasmic cytochrome c levels there was no increase in caspase 3 activity (Figure 4B). Similarly Baf1A treatment was not associated with the cleavage of ICAD, a target of activated caspase 3 (Figure 1C). We conclude that caspase-dependent cell death does not contribute to cell loss by exposure to hypoxia-Baf1A. These results are consistent with previous studies by Zhang et al. [36]. To determine whether hypoxia- Baf1A results in calpain activation we measured the cleavage products of the calpain substrate α-fodrin. Calpains cleave α-fodrin into 150 and 145 kDA products. Treatment of hypoxic cultures with Baf1A resulted in the generation of substantial 145 kDa cleavage products by 18 hrs of treatment (Figure 4D).

### Baf1A reduces tumor growth in a xenograft model

**Figure 5:**
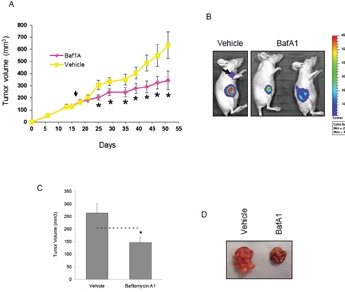
Bafilomycin 1A reduces the growth of both MCF7 and MDA-MB-231–luc xenograft tumors Xenografts were generated in nude mice using (5 x 10^6^) MDA-MB-231 cells stability transfected with luciferase. Once the tumors reached a volume of 100 mm3 the mice were treated (i.p.) every other day with 1.0 mg/kg Baf1A. The progression of tumor growth in vehicle and Baf1A treated mice is shown in (A). Arrow points to start of Baf1A treatment. In (B) MDA-MB-231-luc tumor cell viability was assessed at day 54 by detection of chemiluminescent signal using IVIS imaging system. Arrow points to a metastatic tumor in a vehicle control animal. In (C), MCF7 generated xenografts were treated with 0.1 mg/kg Baf1A intratumorally every other day for 23 days. Final tumor volumes are shown. Dotted line indicates average tumor volume at the start of Baf1A treatment. Representative excised tumors are shown in (D). Data are means ± SEM, n ≥ 5 animals per group. * Significantly different (*p* < 0.05) from vehicle treated animals.

Next we used a xenograft model to investigate whether Baf1A treatment reduced tumor growth. Xenografts were generated using MDA-MB-231 (Figure 5A) and MCF7 cells (Figure 5C). Mice with tumors ≥ 100mm^3^ were treated by i.p. every other day with 1 mg/kg Baf1A or vehicle (1% ETOH). As shown in figure 5A and B, tumor growth was significantly reduced by Baf1A treatment at day 7 and the inhibition continued for the duration of the study. Baf1A treated tumor volumes were on average 50% smaller at the end of the study relative to vehicle-treated control animals. In this study Baf1A reduced the rate of tumor growth but it did not cause tumor regression (tumor volumes at day 50: vehicle 4.0 fold (*p* = 0.0077, n = 8) and Baf1A 2.0 fold (*p* = 0.031, n = 10) over tumor volume at day 1 of Baf1A treatment. To determine whether we could induce tumor regression by a different delivery method that circumvented the vasculature, we delivered Baf1A to MCF7 generated xenografts by intratumoral injection. As shown in figure 5C, tumor volumes were reduced 45% (*p*<0.05; n = 10) by Baf1A treatment compared to vehicle. However, and more importantly, Baf1A treatment caused significant tumor regression with 25% smaller tumor volumes than at the onset of treatment. In comparison vehicle treated tumors grew 138% larger over the same treatment period.

### MAPK pathways are activated by Baf1A treatment

**Figure 6:**
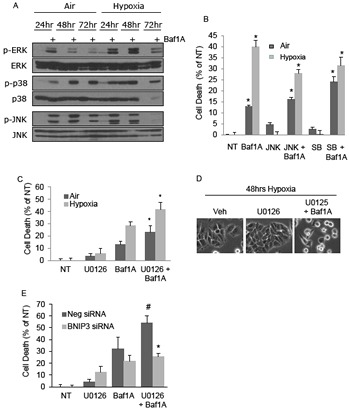
The MAPK signaling pathway contributes to and protects from bafilomycin 1A induced cell death Cells were treated with Baf1A in air or hypoxia for times indicated and the level of ERK, p38 and JNK phosphorylation determined by western blot analysis (A). In (B), cell death was determined in aerobic and hypoxic MCF7 cells treated with either the JNK inhibitor, SP600125 (10 μM), or with the p38 inhibitor, SB203580 (SB) (10 μM) in the presence and absence of Baf1A (p < 0.05; * significantly different than non-Baf1A treated counterparts.) To determine the effects of ERK activation on Baf1A induced cell death, aerobic and hypoxic MCF7 cells were treated with the ERK inhibitor, U0126 (10 μM) in the presence and absence of Baf1A. Cell death is shown in (C) (p < 0.05; * significantly different then Baf1A treated counterparts) and cell morphology is shown in (D). In (E) MCF7 cells were treated with random or Bnip3 specific siRNA and the percent cell death determined during hypoxia in the presence of U0126, Baf1A and U0126+Baf1A (*p* < 0.05; # significantly different then Baf1A; * significantly different then neg siRNA/U0126/Baf1A treated cultures. Data are means ± SEM., n = 5.

Our results show that Baf1A treatment promotes tumor cell death and can regress tumor volumes. As an approach to investigate a more aggressive Baf1A-mediated treatment we investigated the effects of Baf1A on survival MAP kinases. As shown in Figure 6A, the phosphorylation levels of ERK, p38 and JNK were all increased by Baf1A treatment in both aerobic and hypoxic cells. Notably, the effects of Baf1A on p38 and JNK were not dependent on oxygen availability. However, ERK phosphorylation was increased in the hypoxia-Baf1A group relative to normoxia in the absence and presence of Baf1A. To determine whether MAPK activity interfered with Baf1A-induced cell death we used selective inhibitors of each of these pathways and quantified cell death by LDH release. When JNK or p38 were inhibited in aerobic cultures with or without Baf1A there were trends for increased cell death (Figure 6B). In contrast inhibition of either kinase in the hypoxia-Baf1A group was associated with significantly reduced cell death. This data suggests that p38 and JNK may enhance Baf1A toxicity. Next we examined cell death in the presence of ERK1/2 inhibitor, U0126. As shown in Figure 6C and D, ERK inhibition alone increased the level of cell death in both aerobic and hypoxic cultures. ERK inhibition in the presence of Baf1A synergistically increased cell death in both culture conditions. Baf1A and U0126 mediated cell death was reduced when cells were treated with Bnip3 specific siRNA suggesting that Bnip3-mediated cell death is compromised by ERK1/2 activation during Baf1A treatment (Figure 6E). These results suggest that ERK activation is a survival response of these cells to Baf1A treatment, an effect that may be mediated by pH.

**Figure 7:**
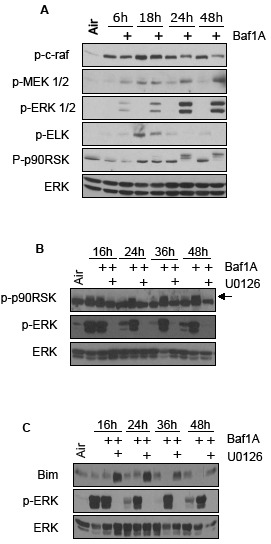
Cytoprotective pathways activated by Baf1A Phosphorylation levels of the c-Raf, Mek1/2, ERK1/2 signaling pathway in hypoxic MCF7 cells exposed to Baf1A for times indicated. Elk and p90RSK are downstream targets of ERK1/2 (A). The effects of the ERK1/2 inhibitor U0126 on Baf1A induced p90RSk hyperphosphorylation and Bim protein degradation is shown in (B and C). Arrow points to hyperphosphorylated p90RSK. All results are representative of at least 3 experiments.

To better understand the cytoprotective action of ERK in the context of Baf1A-induced cell death, we exposed MDA-MB-231 cells to hypoxia in the presence and absence of Baf1A and quantified c-raf-MEK1/2- ERK1/2 signaling. As shown in Figure 7A, c-raf protein phosphorylation levels declined with increasing hypoxia-Baf1A exposure whereas there was a parallel increase of MEK1/2 phosphorylation. Analysis of proteins downstream of ERK1/2 revealed a reduction in ELK phosphorylation and hyperphosphorylation of p90RSK (Fig 7A). Interestingly, p90RSK is known to increase the activity of the Na^+^/H^+^ exchanger by phosphorylation, an effect that may reduce the intercellular acidosis caused by the loss of V-ATPase activity [37]. Inhibition of ERK1/2 with U0126 reversed the Baf1A mediated hyperphosphorylation of p90RSK (Figure 7B); an effect predicted to inhibit protection by p90RSK. Similarly pro-apoptotic Bim is targeted for ubiquitination and proteasomal degradation by ERK1/2 phosphorylation. We found that Bim degradation during hypoxia-Baf1A treatment was also prevented by U0126 treatment. Therefore ERK inhibition is predicted to augment BafA-mediated cell death in hypoxic tumor cells by blocking multiple pathways associated with acidosis and intrinsic apoptosis (Figure 7C).

### ERK inhibition augments the anti-tumor actions of Baf1A

**Figure 8:**
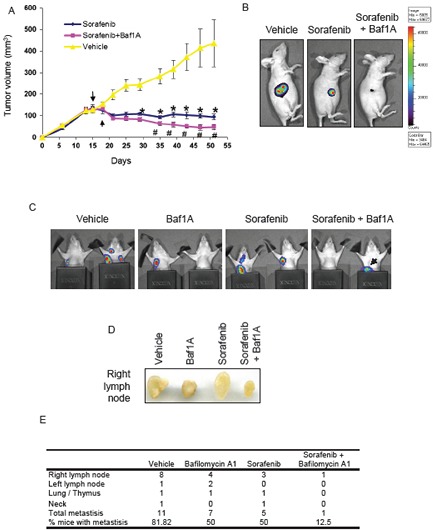
Tumor growth and metastasis are reduced by bafilomycin 1A combined with ERK inhibition Xenografts were generated in nude mice using (5x10^6^) MDA-MB-231 cells stability transfected with luciferase. Once the tumors reached a volume of 100 mm3 the mice were treated (i.p.) every other day with 1.0 mg/kg Baf1A and everyday with the ERK inhibitor, sorafenib (40 mg/kg) by gavage. The progression of tumor growth in vehicle, sorafenib and sorafenib + Baf1A treated mice is shown in (A). In (B) representative images of tumor cell viability assessed on day 50 by detection of chemiluminescent signal using IVIS imaging system. Tumor metastases were visualized by detection of chemiluminescent signal using IVIS imaging system (C). Black metal shields were placed around the lower extremity to prevent signal from the primary tumor. Black arrow is primary tumor signal bleed through and not a metastatic signal. In (D), representative image of excised right lymph nodes. Quantification of metastases and locations is shown in (E). Data are means ± SEM. n ≥ 5 animals per group. *p* < 0.05, * significantly different then sorafenib alone; # significantly different then sorafenib alone.

Next we were interested in determining the therapeutic potential of combinatorial treatment of Baf1A with the ERK inhibitor sorafenib. Sorafenib is an inhibitor of tyrosine kinases including Raf and is currently approved clinically for the treatment of renal and liver cancers [38, 39]. As shown in supplemental Figure 1 we found that treatment of MDA-MB-231-luc cells with 0.5 μM of sorafenib was sufficient to reduce ERK phosphorylation caused by hypoxia and Baf1A. To determine the effect of sorafenib on tumor progression we generated MDA-MB-231-luc xenografts in nude mice. Mice with tumor volumes > 100mm^3^ were treated with either sorafenib alone or combined with 1mg/kg Baf1A i.p. as described in Methods. As shown in Figure 8A and B, sorafenib treatment significantly blocked tumor growth at all times relative to vehicle treated control mice. Tumor volume in the vehicle group increased in a linear manner reaching a final volume ~ 4x the starting size. In contrast tumor volumes in sorafenib-treated animals were not significantly different to those observed at the start of treatment. It should be noted that sorafenib prevented tumor growth but did not cause substantial tumor regression. In contrast to sorafenib alone, the combination of sorafenib and Baf1A resulted in a significant reduction in tumor volume by treatment day 12 and continued to decrease tumor volume by a factor of 40% of the starting tumor volume by day 55. This data indicates that the combination treatment of Baf1A and sorafenib not only prevents tumor growth but promotes tumor regression.

Next we determined the effects of combinatorial Baf1A and sorafenib treatment on tumor metastasis using the same MDA-MB-231-luc xenografts. Metastasis were determined by non-invasive luciferase as described in Methods. Examples of metastatic localization and the effect of drug treatment are shown in Figure 8C and D. Metastases were found in the right and left lymph node, the neck and the area of the lungs/thymus. Metastatic quantification revealed that 82% of vehicle treated mice exhibited 1 or more metastases. The number of mice with metastasis was significantly reduced by both Baf1A (5 out of 10 animals) and sorafenib (5 out of 10 animals) treatments. The number of metastasis was further significantly reduced (*p* < 0.05) by the combined treatment with only 1 of 8 mice displaying metastasis in the sorafenib + Baf1A group (Figure 8E).

## DISCUSSION

Our results show that treatment of hypoxic MCF7 and MDA-MB-231 breast cancer cells with the V-ATPase inhibitor Baf1A activated Bnip3-dependent cell death. The effect was mimicked by knockdown of V-ATPase with a selective siRNA and blocked by a Bnip3-selective siRNA. A caspase independent programmed death pathway that involves mitochondrial permeability transition was implicated and is consistent with our previous results and results from other laboratories [32, 33]. We hypothesize that at least part of the mechanism of cell death involves Bnip3 activation by intracellular acidosis. Baf1A treatment also activated MAPK promoting survival and this was countered by a combination treatment of Baf1A and ERK 1/2 inhibition. The *in vitro* effects were well replicated *in vivo* in breast cancer xenografts. Baf1A treatment significantly blocked tumor growth and even cause regression when delivered directly into the tumor. Combination therapy with Baf1A and the MAPK inhibitor sorafenib optimally inhibited tumor growth, conferred tumor regression and reduced metastasis. These are the first studies to show activation of the Bnip3 death pathway by modulation of proton flux and its possible relevance to the treatment of breast and possibly other cancers.

Hypoxic tumors are known to activate elaborate defense mechanism specifically to evade Bnip3–mediated death. In pancreatic adenocarcinoma and colorectal cancer Bnip3 transcription is prevented by hypermethylation of the Bnip3 promoter [40, 41]. In glioblastoma and non-small lung carcinomas Bnip3 protein is sequestered in the nuclei where it may actually block apoptosis by suppressing transcription of the apoptosis-inducing factor (AIF) and death receptor 5 (DR5) genes [42, 43]. Our studies suggest a third mechanism whereby intracellular acidosis is avoided and Bnip3 in hypoxic regions is maintained in a latent state through the aggressive extrusion of protons by the V-ATPase pump. Hypoxic MCF7 and MDA-MB-231 cells remained fully viable during 72 hrs of severe hypoxia despite the expression of Bnip3. Loss of cell viability and Bnip3-dependent death was only evident after treatment with Baf1A. Knock-down of Bnip3 with siRNA reversed Baf1A induced cell death. These results are consistent with our previous result that described Bnip3 death pathway mediated by hypoxia-acidosis in cardiac myocytes and confirm a requirement of increase intracellular acidosis to activate cell death by Bnip3 [31, 32]. The results are also consistent with other reports that elevated Bnip3 alone does not induce death or autophagy in hypoxic cells [44] [45].

Baf1A treatment was found to increase the phosphorylation levels of the mitogen activated protein kinase (MAPK) signaling pathways including JNK, p38 and ERK. The ras/raf/ERK signaling pathway is of particular interest since one or more mutations in proteins associated with this pathway has been found in many types of cancers [46]. Unlike JNK and p38 that may assist in Baf1A induced cell death, ERK activation was protective. Consequently ERK inhibition synergized with Baf1A to enhance toxicity and promote cell death. ERK was found to phosphorylate the proapoptotic protein Bim targeting it for degradation, and caused increased phosphorylation of p90RSK. P90RSK has been shown to positively regulate the activity of the Na^+^-H^+^ exchanger an activity that may contribute to pH regulation [37, 47]. Therefore ERK activation exerts a dual survival response to block Bnip3-mediated death by enhancing pH regulation and blocking Bim-mediated death by proteosomal targeting.

To determine therapeutic potential of Baf1A on tumor growth *in vivo* we used xenograft models of moderately aggressive non-metastatic breast cancer (MCF7) and highly aggressive metastatic breast cancer (MDA-MB-231). Baf1A reduced the rate of tumor growth by ~50% in each of the xenograft models. Importantly, we observed no toxic side effects of Baf1A at the concentration used. This is in agreement with other studies that have examined Baf1A on tumor growth [48, 49]. The therapeutic effects of Baf1A were significantly enhanced by the addition of the ERK inhibitor sorafenib.

Tumor metastasis is a critical factor in cancer patient morbidity and is becoming increasingly linked to extracellular acidification [50-52]. In metastatic breast cancer cells both migration and invasiveness are increased when the cells are exposed to acidic conditions [53, 54]. Inhibition of the V-ATPase with Baf1A is able to reduce the migration and invasiveness of cancer cells *in vitro* suggesting a direct role for the V-ATPase in tumor metastasis [20]. In rapidly dividing metastatic breast cancer cells the V-ATPase is localized to the plasma membrane whereas in less aggressive breast cancer cells the V-ATPase is localized to the lysosomes [20]. Using the highly aggressive and metastatic breast cancer cell line MDA-MB-231-luc, we observed a 50% reduction in the number of metastatic tumors in the presence of Baf1A alone that was further reduced by the combination of Baf1A and sorafenib. It has been proposed that proton extrusion by the V-ATPase produces a permissive environment for tumor cell metastasis by regulating protease digestion of the extracellular matrix, by increasing tumor cell growth and migration, and by increasing angiogenesis [55-57] [58].

Breast cancer treatment is largely determined by the stage and characteristics of the tumor. Treatment strategies include mastectomy and lumpectomy usually followed by radiation, chemo and hormone therapy. In most of these cases the acidic and hypoxic tumor microenvironments negatively impact the success of these treatments by inducing radiation and chemotherapy resistance and by selecting cells with enhanced metastatic phenotypes [59-61]. However, the majority of breast cancer cells and cancers cells in general may hold the keys to their own demise in the expression of the hypoxia regulated apoptotic protein Bnip3. We believe that our results warrant further studies on combination therapy focused on maximal Bnip3 activation in the hypoxic zone, and blockade of compensatory pathways by which the tumor cells evade such death. The targeting of hypoxic tumor cells is particularly exciting because these are the cells that confer resistance to radiation and chemotherapy and may in fact harbor tumor stem cells capable of regenerating the entire tumor [62] [63].

## MATERIALS AND METHODS

RPMI Medium, and Fetal Bovine Serum (FBS) were purchased from Gibco/Life Technologies (Grand Island, NY). All other reagents were purchased from Sigma (St. Louis, MO) unless otherwise noted.

### Cell culture

Human breast cancer cells MCF7, and MDA-MB-231 were purchased from ATCC. MDA-MB-231-lucerifase cells were purchased from Cell Biolabs Incorporated. Cells were maintained in RPMI media supplemented with 10% fetal bovine serum and 1% penicillin/streptomycin.

### Xenografts

All animal protocols were approved and conducted in accordance with institutional guidelines for the care and use of animals. Xenografts were generated using MCF-7 and MDA-MB-231-luciferase human breast cancer cells. Subconfluent cultures where trypsinized and resuspended in cool RPMI and Matrigel (Collaborative Biomedical) at a 1:2 ratio. Xenografts were generated by injecting 0.1 ml of Matrigel/medium mixture containing 5 x 10^6^ cells into the right flank of nude mice (Charles River Laboratory). Tumor volumes were determined by measuring tumor length and width with calipers and the tumor volume determined using the formula L ^x^ W^2^/2. Once the tumor volumes reached 100 mm^3^, the animals were divided into randomly assigned treatment groups receiving either 1) vehicle, 2) 1mg/kg nM Baf1A administered every two days in a volume of 0.1 ml, 3) 40 mg/kg sorafenib administered daily by gavage, or 4) Baf1A + sorafenib administered as described above. In certain experiments mice were injected with 150 mg/kg luciferin and the tumor burden and metastasis visualized using a Xenogen IVIS-200 imaging system (UM Oncogenomics Core facility).

### Hypoxia

Cells were exposed to hypoxia using a BioTrace anaerobic chamber as previously described [32]. Chamber oxygen concentration was continually monitored and maintained at 0.5% oxygen with 5% carbon dioxide. To insure adequate glucose concentrations during hypoxia, cell culture media was supplemented with 10 mM glucose. Cells were lysed under hypoxia using ice-cold hypoxic buffers.

### siRNA treatment

Bnip3 and V-ATPase proteins were knocked down as previously described [64] using protein specific siRNA obtained from Dharmacon (Thermo Scientific). Cells were transfected overnight with 10 nM Bnip3 siRNA, or 20, 50, or 100 nM V-ATPase siRNA or random sequence siRNA using DharmaFECT 4 transfection reagent according to the manufacturer's instructions.

### Cell transfection

Cells were transfected with vectors expressing GFP, Bnip3 or Bnip3 transmembrane deletion mutant using Lipofectamine 2000 (Invitrogen) [32]. After 6 hr the transfection media was removed and the cells imaged 48 hr following Baf1A treatment.

### Caspase 3 activity

Caspase 3 activity was determined using a fluorometric assay from R&D systems, as per the manufacturer's instructions and analyzed using a Victor 1420 Multilabel counter.

### Cell death assays

Cell death was determined by trypan blue extrusion as previously described and by lactate dehydrogenase release using a commercially available kit (Roche).

### Subcellular fractionation

Cells were fractionated as previously described [33]. In brief, after treatment, cells were washed with PBS and suspended in 10 mM Tris, pH 7.4, and 320 mM sucrose, 1 mM EDTA, 1 mM DTT with protease and phosphatase inhibitors and homogenized using a teflon-glass homogenizer. The resulting homogenate was centrifuged at 1,500 x g for 5 min at 4°C to pellet the nuclei and unbroken cells. The supernatant was further centrifuged at 10,000 x g for 20 min at 4°C to pellet mitochondria.

### Western blot analysis

Cells were lysed in RIPA buffer and western blots performed as previously described [64]. Western blot densitometry was determined using Image J software from NIH. Western blot analysis was performed using antibodies to Bnip3 (Abcam), V-ATPase (EMD Millipore), PUMA, NOXA, Bim, Mcl-xl, Bcl-2, Bak (Cell Signaling Technologies), cytochrome c (Pharmingen), ICAD (Santa Cruz Biotechnology), α-fodrin (Chemicon International), phospho-ERK, p38, JNK, MEK1/2, ELK, and p90RSK (Cell Signaling Technologies). Equal protein loading was demonstrated by reprobing the membranes with β-actin (Sigma), VDAC (Biovision), ERK, p38, and JNK (Cell Signaling Technologies).

### Statistical analysis

All data is expressed as mean ± SEM of at least three experiments. Statistical analysis was performed using Social Sciences Statistical Package (SPSS, Inc). Student t-test analysis was used for two-group comparison whereas multigroup analysis was performed using a one-way ANOVA analysis with Bonferroni's correction. *p* < 0.05 was considered to be statistically significant.

## SUPPLEMENTARY FIGURE




