# Supplementary material for: Inhibition of the vacuolar ATPase induces Bnip3-dependent death of cancer cells and a reduction in tumor burden and metastasis
Source: Oncotarget. 2013 Dec 29;5(5):1162–73. doi: 10.18632/oncotarget.1699 (PMC4012732; doi:10.18632/oncotarget.1699)
Supplement: Supplementary file 1 [file oncotarget-05-1162-s001.docx]

Supplementary Figure

MDA-MB-231-luc cells were exposed to Baf1A (10 nM) for times indicated in the presence of vehicle or increasing concentrations ( 0.1, 0.5, 1, 5, 10 μM) of sorafenib. ERK1/2 phosphorylation levels were determined by Western blot analysis. Representative of 3 experiments.
