# Supplementary figures and images for: Inhibition of the vacuolar ATPase induces Bnip3-dependent death of cancer cells and a reduction in tumor burden and metastasis
Source: Oncotarget. 2013 Dec 29;5(5):1162–73. doi: 10.18632/oncotarget.1699 (PMC4012732; doi:10.18632/oncotarget.1699)

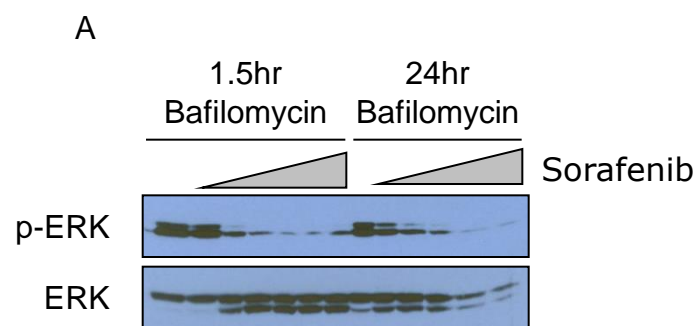

Supplement: Supplementary file 2 [file oncotarget-05-1162-s002.pdf]
